# Supplementary figures and images for: Hepatitis C virus non-structural proteins modulate cellular kinases for increased cytoplasmic abundance of host factor HuR and facilitate viral replication
Source: PLoS Pathog. 2023 Aug 4;19(8):e1011552. doi: 10.1371/journal.ppat.1011552 (PMC10431626; doi:10.1371/journal.ppat.1011552)

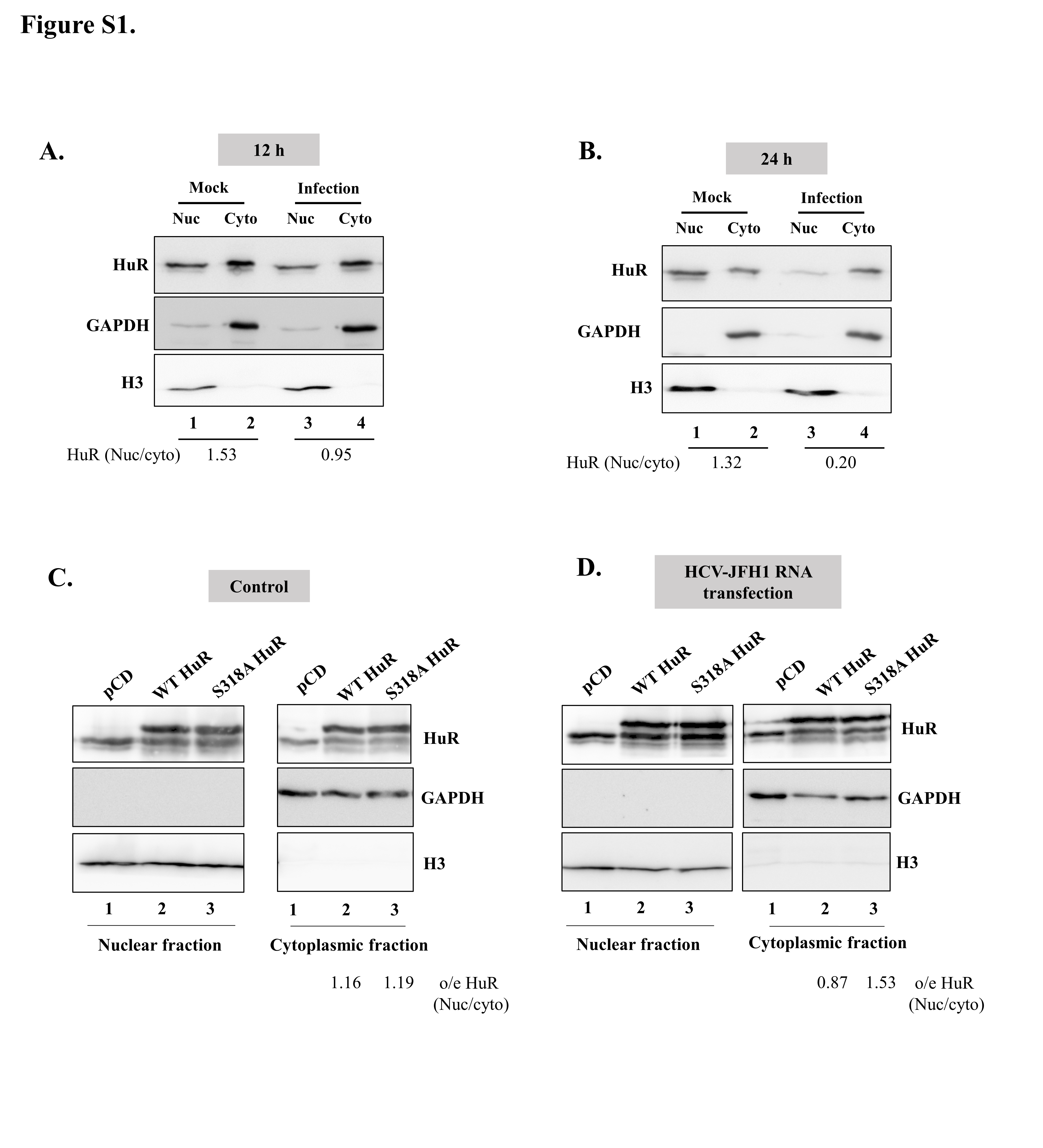

Supplement: S1 Fig — (A, B) Huh7.5 cells were infected with HCV-JFH1 virus and 12h (A) or 24h (B) post infection, cells were harvested, and nuclear cytoplasmic fractionation performed. Western blotting was done for nuclear and cytoplasmic fraction to determine the ratio of HuR in nucleus and cytoplasm. GAPDH was used as a marker for cytoplasmic fraction and Histone H3 as a marker for nuclear fraction. Densitometry was performed and the numbers in bottom indicate Nuclear to cytoplasmic ratio of HuR after normalising with H3 and GAPDH. (C, D) Huh7.5 cells were transfected with vector control (pcDNA3.1), WT HuR overexpression construct or S318A HuR overexpression construct as indicated. 16h post transfection, cells were infected with HCV-JFH1 virus and 48h post infection, cells were harvested, and nuclear cytoplasmic fractionation performed. Western blotting was done for nuclear and cytoplasmic fraction to determine the ratio of HuR in nucleus and cytoplasm. The lower band in HuR blot represents endogenous HuR and the upper band represents overexpressed HuR. GAPDH was used as a marker for cytoplasmic fraction and Histone H3 as a marker for nuclear fraction. Densitometry was performed for overexpressed HuR (o/e HuR) and the numbers in bottom indicate Nuclear to cytoplasmic ratio of o/e HuR after normalising with H3 and GAPDH. (TIF) [file ppat.1011552.s001.TIF]

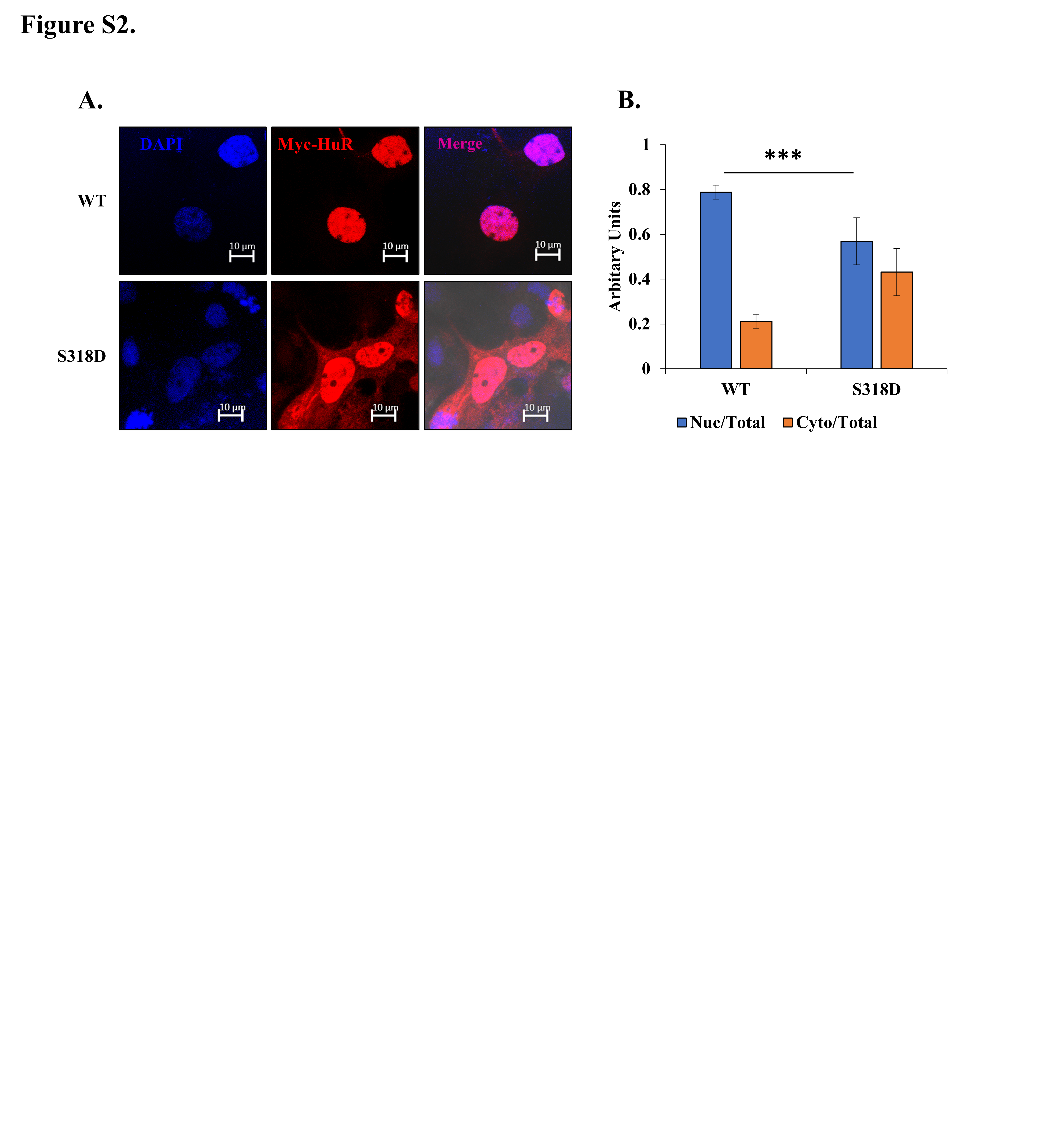

Supplement: S2 Fig — (A) Huh7.5 cells were transfected with either Myc-WT HuR or Myc-S318D HuR and immunofluorescence staining was carried out after 48h using Alexa Fluor conjugated secondary antibodies against Myc (Red). The nucleus was counterstained with DAPI. Scale bar represents 10 μm. (B) Nuclear and cytoplasmic ratio for Myc-HuR was quantified for images in (A) using Zen 2.3 lite software. n = 10. (TIF) [file ppat.1011552.s002.TIF]

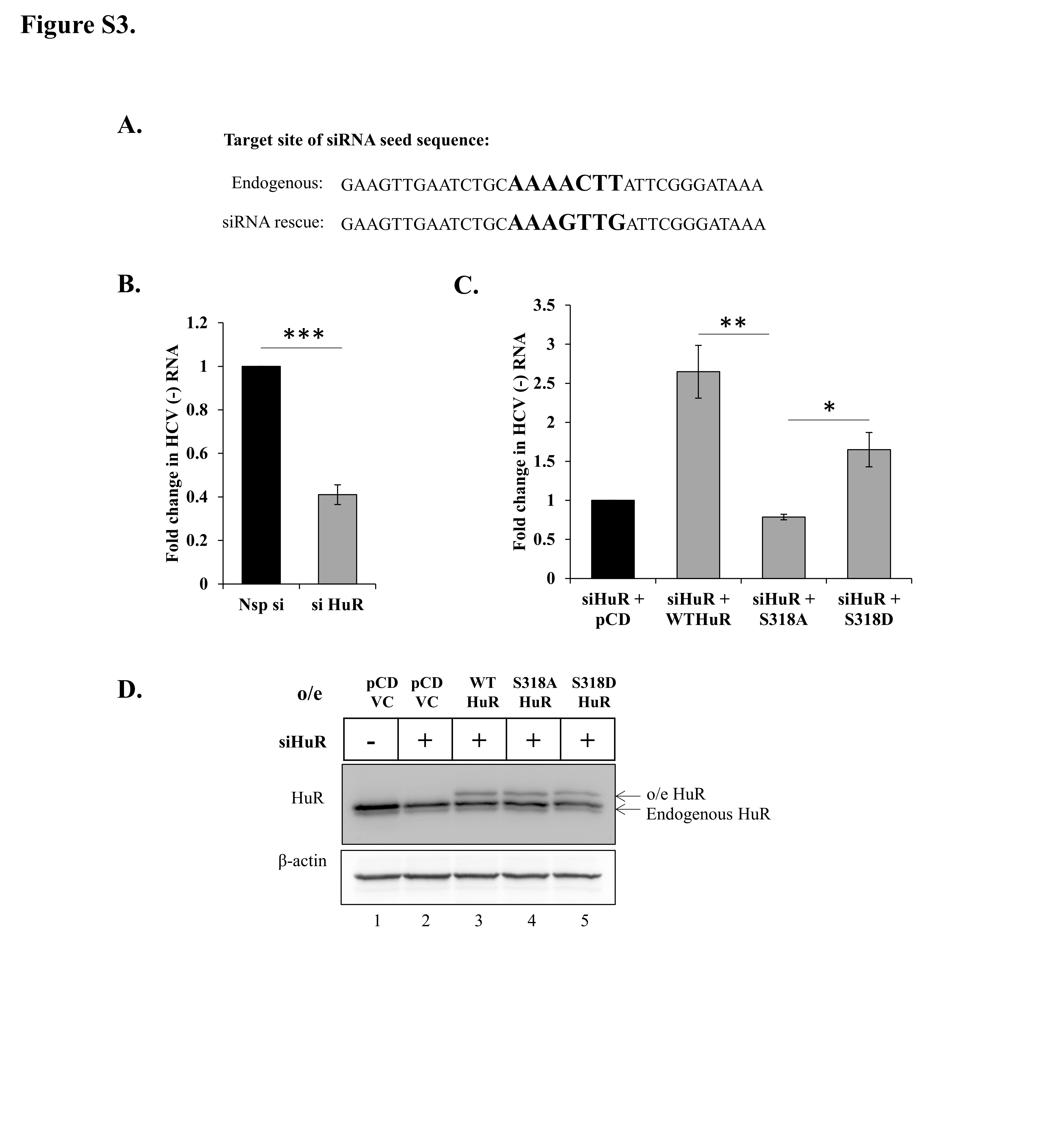

Supplement: S3 Fig — (A) The schematic of the mutations generated in HuR overexpression constructs for siRNA rescue. (B, C) Huh7.5 cells were transfected with either a non-specific siRNA (Nsp si) or siRNA targeting HuR (siHuR) along with the described siRNA rescue HuR overexpression constructs. HCV-JFH1 RNA was transfected after 16-24h of overexpression. 48h post HCV RNA transfection, cells were harvested, and HCV negative strand RNA quantified using real time PCR. (n = 3). (D) Western blotting for HuR was performed following the experiment in (B) and (C). o/e denotes the overexpression of described pCD VC (vector control)/ WT/ mutant HuR. The HuR overexpression constructs are siRNA recue constructs generated in (A). A non-specific siRNA was transfected in lane 1 and siRNA in lanes 2–5. Student t-test was performed for statistical analysis. * = p<0.05, ** = p<0.01, *** = p<0.001. (TIF) [file ppat.1011552.s003.tif]

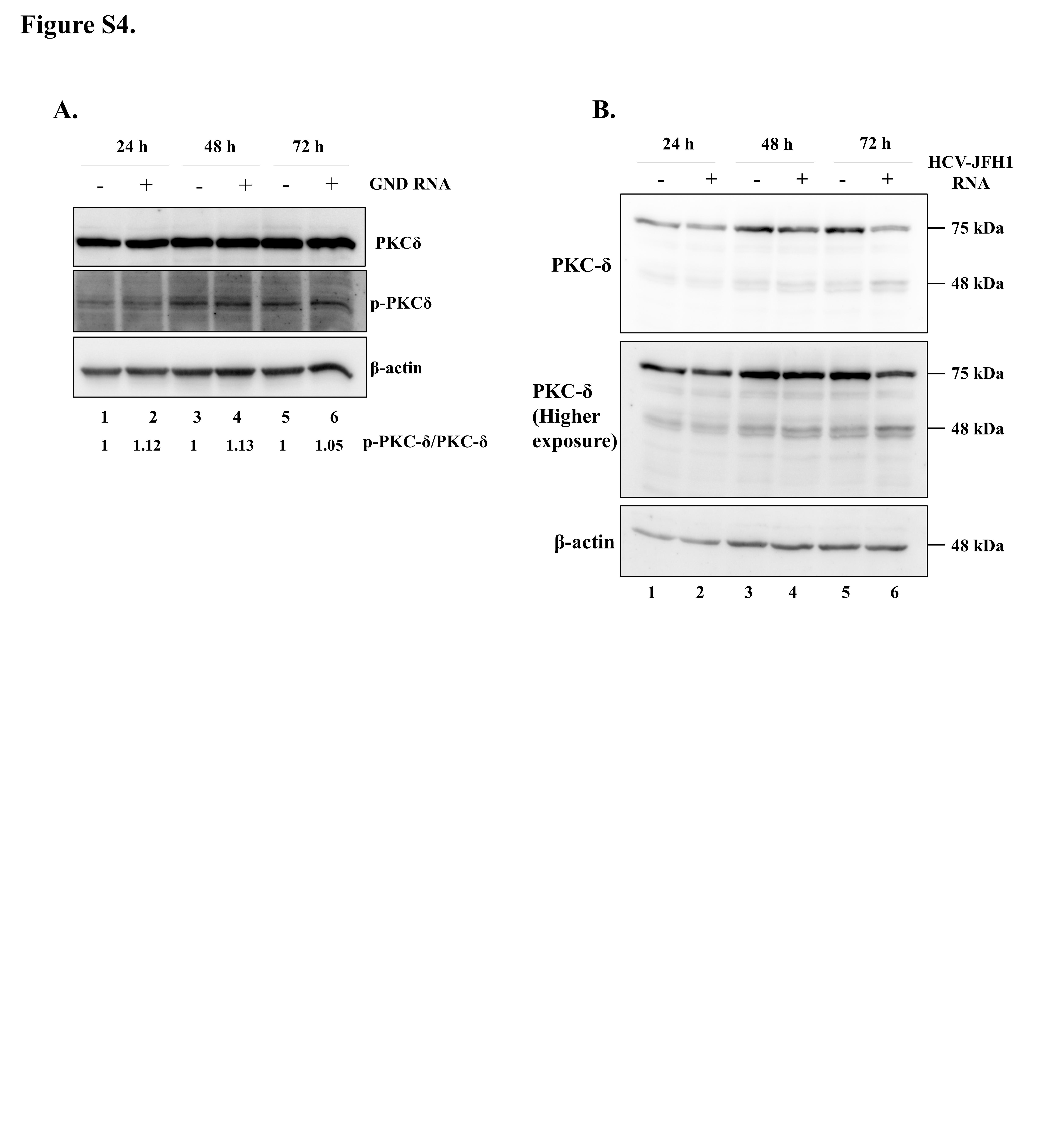

Supplement: S4 Fig — (A) Huh7.5 cells were transfected with pSGR-JFH1/Luc-GND RNA and cells harvested at indicated time-points. Western blotting was done with anti-PKC-δ, anti-p-PKC-δ and anti-actin antibodies. Appropriate HRP-conjugate secondary antibodies were used. (B) Huh7.5 cells were transfected with HCV-JFH1 RNA and cells harvested at indicated time-points. Western blotting was done with anti-PKCδ, and anti-actin antibodies. Appropriate HRP-conjugate secondary antibodies were used. (TIF) [file ppat.1011552.s004.tif]
